# The Impact of a Mobile Support Group on Distress and Physical Activity in Breast Cancer Survivors: Randomized, Parallel-Group, Open-Label, Controlled Trial

**DOI:** 10.2196/47158

**Published:** 2023-08-07

**Authors:** Miyeon Jung, Sae Byul Lee, Jong Won Lee, Yu Rang Park, Haekwon Chung, Yul Ha Min, Hye Jin Park, Minsun Lee, Seockhoon Chung, Byung Ho Son, Sei-Hyun Ahn, Il Yong Chung

**Affiliations:** 1 Lee Business School, University of Nevada Las Vegas Las Vegas, NV United States; 2 Department of Surgery, University of Ulsan College of Medicine, Asan Medical Center Seoul Republic of Korea; 3 Department of Biomedical Systems Informatics, Yonsei University College of Medicine Seoul Republic of Korea; 4 Swallaby Inc Seoul Republic of Korea; 5 College of Nursing, Kangwon National University Chuncheon Republic of Korea; 6 Department of Psychiatry, University of Ulsan College of Medicine, Asan Medical Center Seoul Republic of Korea

**Keywords:** breast neoplasms, mental distress, mobile apps, mobile health intervention, mobile phone, physical activity, randomized controlled trial, RCT, support group, survivorship, telemedicine

## Abstract

**Background:**

While mobile health apps have demonstrated their potential in revolutionizing health behavior changes, the impact of a mobile community built on these apps on the level of physical activity and mental well-being in cancer survivors remains unexplored.

**Objective:**

In this randomized controlled trial, we examine the effects of participation in a mobile health community specifically designed for breast cancer survivors on their physical activity levels and mental distress.

**Methods:**

We performed a single-center, randomized, parallel-group, open-label, controlled trial. This trial enrolled women between 20 and 60 years of age with stage 0 to III breast cancer, an Eastern Cooperative Oncology Group performance status of 0, and the capability of using their own smartphone apps. From January 7, 2019, to April 17, 2020, a total of 2,616 patients were consecutively screened for eligibility after breast cancer surgery. Overall, 202 patients were enrolled in this trial, and 186 patients were randomly assigned (1:1) to either the intervention group (engagement in a mobile peer support community using an app for tracking steps; n=93) or the control group (using the app for step tracking only; n=93) with a block size of 10 without stratification. The mobile app provides a visual interface of daily step counts, while the community function also provides rankings among its members and regular notifications encouraging physical activity. The primary end point was the rate of moderate to severe distress for the 24-week study period, measured through an app-based survey using the Distress Thermometer. The secondary end point was the total weekly steps during the 24-week period.

**Results:**

After excluding dropouts, 85 patients in the intervention group and 90 patients in the control group were included in the analysis. Multivariate analyses showed that patients in the intervention group had a significantly lower degree of moderate to severe distress (B=–0.558; odds ratio 0.572; *P*<.001) and a higher number of total weekly step counts (B=0.125; rate ratio 1.132; *P*<.001) during the 24-week period.

**Conclusions:**

Engagement in a mobile app–based patient community was effective in reducing mental distress and increasing physical activity in breast cancer survivors.

**Trial Registration:**

ClinicalTrials.gov NCT03783481; https://classic.clinicaltrials.gov/ct2/show/NCT03783481

## Introduction

Cancer survivors often face psychological distress following their diagnosis, with anxiety and depression being prevalent among them. Approximately 18% of cancer survivors experience anxiety, while depression impacts around 12% of individuals who have had cancer [1]. In a systematic review focused on breast cancer survivors, the prevalence of mood disorders was found to vary from 22% to 60%, while the range for depression was between 20% and 65% [2]. Depression is recognized as a considerable health issue among breast cancer survivors and is linked to a diminished quality of life related to health [3]. The improved outcomes of cancer treatment in recent times have sparked a growing interest in the quality of life experienced by individuals who have survived cancer [4]. International guidelines suggest that it is crucial to regularly address mental distress starting from the moment of cancer diagnosis, as it has a significant impact on the overall quality of life for individuals who have survived cancer [5,6].

The management of mental distress in cancer survivors includes a variety of interventions, including psychosocial or pharmacologic support, complementary therapies, and exercise [5]. The American Cancer Society highlights compelling evidence that endorses the positive impact of physical activity on enhancing the quality of life and managing mental well-being in individuals with cancer [7]. The panel of the National Comprehensive Cancer Network recommends moderate or vigorous exercise [8]. Furthermore, to avoid a sedentary lifestyle, the panel recommends starting with at least 20 minutes of light activity (eg, slow walking) per week, especially for inactive cancer survivors, as even light exercise can improve physical functioning and is safe for cancer survivors [8]. Nonetheless, the availability of resources and programs for physical activity is frequently constrained by factors such as time constraints, limited workforce, and inadequate insurance coverage. Consequently, additional research is required to assess the efficacy and cost-effectiveness of various behavior change programs targeting cancer survivors.

In recent times, there has been an increasing inclination toward employing mobile apps as a means to deliver behavioral health interventions. To assess the effectiveness of mobile apps in enhancing health behaviors, a systematic review was conducted [9]. Although the overall evidence did not strongly support the notion that mobile apps improve health behaviors, certain behaviors showed noteworthy results. For example, in the context of physical activities such as walking, 3 of 7 (43%) studies reported positive changes [10,11]. Additionally, all 3 (100%) studies indicated a positive impact of mobile apps on reducing sedentary behavior [11-13].

Moreover, the effectiveness of mobile health (mHealth) interventions in improving health outcomes was examined among cancer survivors. An additional systematic review, examining 16 studies that investigated the impact of mHealth interventions on improving physical activity levels among cancer survivors [14], revealed that 8 studies reported a significant increase in step counts and duration of physical activity [15-22]. The intervention components commonly used in these studies included wearable activity trackers, coaching, websites, and SMS text messages. While mHealth interventions have shown promise in promoting physical activity, the impact of a mobile community built on apps on the level of physical activity and mental well-being in cancer survivors remains unexplored [14]. In this randomized controlled trial, we investigate the effects of a mobile phone–based peer community for cancer survivors on promoting physical activity and alleviating mental distress, specifically in breast cancer survivors.

## Methods

### Trial Design and Patients

We conducted a single-center, parallel-group, open-label, randomized controlled trial at Asan Medical Center (Seoul, Republic of Korea). Inclusion criteria were female breast cancer survivors aged 20 to 60 years with a stage 0-III disease based on the 7th edition of the American Joint Committee on Cancer Staging Manual and Eastern Cooperative Oncology Group Performance Status 0. Older patients were excluded from the study due to potential challenges they may face in using smartphone apps and reading small fonts, as required by the study protocol. Eligible participants were those capable of using their own smartphones compatible with the Android-based app WalkON (Swallaby Co, Ltd), which is a freely available app that was modified for this study [23]. Patients who had a recurrence, metastasis, severe medical conditions, or scheduled adjuvant chemotherapy were excluded. Patients were enrolled only after breast cancer surgeries while they were hospitalized. Due to the nature of the study, which involved educational sessions on the app, enrollment took a considerable amount of time, so all patients completed their registration during their hospitalization. Patients who were expected to have omitted chemotherapy based on the results of preoperative pathologic examinations and intraoperative frozen sections were enrolled in the study. However, some patients had their staging upstaged, such as the discovery of lymph node metastasis in the final pathologic exams after discharge, leading to the decision to undergo adjuvant chemotherapy. These patients were subsequently excluded from the study after registration. After enrollment, interventions were performed regardless of adjuvant therapies, except for adjuvant chemotherapy, as physical activities could often be limited due to cytotoxic side effects in these patients.

**Figure 1:**
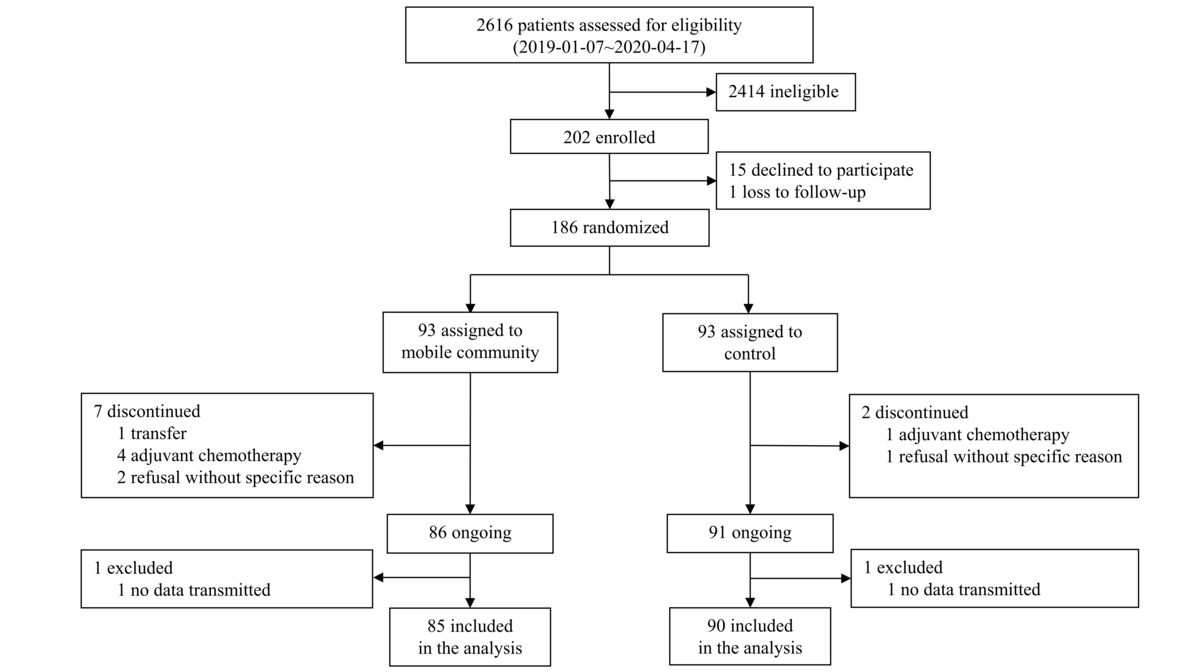
Study enrollment flow chart.

Between January 7, 2019, and April 17, 2020, we consecutively assessed 2616 patients with breast cancer who underwent surgery (Figure 1). Out of the total, 1578 patients were excluded from the study due to meeting the exclusion criteria, 403 patients could not be reached during their hospital stay. A total of 202 patients were enrolled in this study; after enrollment, 15 patients withdrew consent due to inconvenience during the education session on app usage (n=14) and a smartphone error after downloading the app (n=1), and 1 patient was lost to follow-up. The remaining 186 patients were randomly assigned to the intervention group (n=93) and the control group (n=93). In the intervention group, 7 patients discontinued the intervention due to hospital transfer (n=1), adjuvant chemotherapy (n=4), or refusal without specific reason (n=2). In the control group, 2 patients discontinued the intervention due to adjuvant chemotherapy (n=1) or refusal without specific reason (n=1). A total of 177 patients were intended to be analyzed; however, at the completion of the study, 1 participant per group was excluded because they did not send any data on either the distress questionnaire or daily step counts through the mobile app. Finally, 85 patients in the treatment group and 90 patients in the control group were included in the analysis.

### Interventions

The intervention in this study was the use of the breast cancer survivor mobile community function on the WalkON app, which is an activity-tracking app that was previously introduced in our prospective feasibility study [24]. In the mobile community, patients could check their daily step count, share it with other community members, and check their real-time rank based on the daily step counts. Additionally, patients received messages that encouraged physical activity on a weekly basis and could post their messages for other members as well. The patients were instructed to join an existing community that had been established in a previous nonrandomized, prospective, interventional study (ClinicalTrials.gov NCT03190720) conducted at our hospital in order to ensure that all patients, including those that were among the first few patients to be enrolled, were immediately incorporated into an active community with multiple members [23]. Patients in the control group were only able to check their own daily step counts on the WalkON app and not use the mobile community function. All the patients were told to take their smartphones with them when they walked.

### Data collection

The patients’ clinical information was collected from the electronic medical records of the hospital. Clinical data included age at diagnosis, marital status, education, occupation, comorbidity, past episodes of depression, and breast cancer treatments. We also collected 2 types of out-of-hospital data: daily step counts from the WalkON app and app-based self-reported distress questionnaire. During the app registration process, patients received anonymous IDs from researchers, and their information was entered into an external server. As for the self-reported distress questionnaire, all patients in the intervention group and the control group were instructed to fill out a questionnaire on the Distress Thermometer (DT) on a weekly basis. DT (see Figure a in Multimedia Appendix 1), which was developed by the National Comprehensive Cancer Network, is a freely available questionnaire that can be used to monitor the level of mental distress in cancer survivors [25]. The DT is recorded based on an 11-point visual analog scale ranging from 0 (no distress) to 10 (extreme stress). In this study, we used a validated version of app-based DT that was reported to be significantly correlated with the original paper–based DT in a previous study [26]. The daily step counts were automatically collected in the app, in which a pull-to-refresh action sent a weekly bundle of data on daily step counts, time, and user ID to a remote server (Figure b and c in Multimedia Appendix 1). To enhance the data collection rate, push alarms were sent to all patients’ smartphones from Sunday to Tuesday every week. Participants were able to post messages in the chat window and also leave comments (Figure d in Multimedia Appendix 1).

### Procedures

Throughout the duration of the hospital stay after breast cancer surgery, a researcher conducted screenings and maintained contact with the patients. After enrollment, the WalkON app was downloaded to the patients’ smartphones. Patients received instructions on how to use the app, fill out DT questionnaires, and check daily step counts. Patients were excluded from the study if they were recommended to undergo adjuvant chemotherapy based on the postoperative pathological reports or genomic assay results at the first visit to the clinic (2 to 3 weeks after discharge). Except for the abovementioned cases, the remaining patients were randomly assigned to either the control or intervention group, following a 1:1 allocation ratio. Those in the intervention group were subsequently registered on the mobile community by a clinical research assistant. After a period of 3 weeks, the same research assistant contacted the patients to inquire about any technical difficulties they encountered while using the app.

### Outcomes

The prespecified primary outcome was the rate of moderate to severe distress (DT score ≥5) at a time frame of 24 weeks. For analysis, we transformed the weekly DT scores into a binary variable at the cutoff value of 5, constructed a panel data set with the binary outcome variable for 24 weeks, and then used a logit model with control variables. The prespecified secondary outcome was the total number of weekly step counts collected during 24 weeks. We constructed a panel data set with the weekly step counts for 24 weeks and then used a regression model with control variables.

### Sample Size

The sample size was calculated based on the previous feasibility study that investigated the rate of distress according to the use of mobile community intervention [24]. Based on the results of the regression analysis, it was found that joining the mobile community significantly reduced distress. The proportions of moderate to severe distress exceeding 4 of DT were 1.81% (2/110) in the mobile community group and 13.11% (27/206) in the control group, respectively. Considering a 2-sided 5% significance level and 80% power, it was calculated that a decrease of 11.3% in the probability of exceeding 4 of DT would require a total sample size of 168 individuals. Considering a dropout rate of 20% during the study, it is estimated that a total of 202 patient registrations would be necessary.

### Randomization and Masking

A researcher who was not involved in subject enrollment generated a randomization sequence using a computer, employing a block size of 10 without stratification. The researchers responsible for subject enrollment remained blinded to the allocation sequence until the moment of allocation. Both the patients and researchers were not blinded at the time of allocation, as the intervention necessitated active participation in the mobile community. However, during follow-up visits at the outpatient clinic, the attending researchers were blinded to the group allocation. Out-of-hospital data collected through the app were automatically transmitted to the laboratory, where other researchers conducted the analysis.

### Statistical Methods

Clinicopathologic characteristics were described with absolute and relative frequencies. Continuous (eg, age at diagnosis) and categorical variables (eg, marital status, education, occupation, comorbidity, past episode of depression, and breast cancer treatments) were compared between the 2 groups using the Student *t* test and chi-square test.

To estimate the effect of mobile community engagement, the primary outcome (moderate to severe distress) was analyzed by a multivariate logit model using a panel data set of individual patients’ weekly DT scores. For the logit model, we controlled for age at diagnosis, marital status, education level, occupation, comorbidity, previous depression, and breast cancer treatments. Following the econometric philosophy, even if the variables are not significant, including all possible variables as control variables can increase the variance of the coefficients but reduce bias. Therefore, with the aim of minimizing bias, we controlled for all possible variables. The dependent variable was the binary variable indicating whether the patient had a DT score higher than or equal to 5 in a given week, and the independent variable was mobile community engagement [27]. We estimated the logit model for the panel data sets constructed for the total observation period of 24 weeks (time frame of 6 months). The secondary outcome was analyzed by a multivariate generalized linear regression model for the panel data sets constructed for the total observation period of 24 weeks (time frame of 6 months). For the weekly step counts, the Shapiro-Wilk test confirmed an asymmetric distribution of the dependent variable. Considering the characteristics of our dependent variable (step count) and the issue of overdispersion, we performed the Generalized Linear Model using the glm function in R with the “quasipoisson” family option.

Sensitivity analyses were conducted with a dependent variable of DT scores higher than or equal to 3 (mild to severe distress) instead of 5 and panel data sets that were constructed for the total observation period of the past 4 weeks instead of the total 24 weeks. Although the study period was relatively short (6 months), in order to examine the prolonged effects of the mobile community on physical activities and mental distress, our analysis incorporated the data from the past 4 weeks. All analyses were conducted using R Software (version 4.2.2, R Foundation for Statistical Computing), and *P* values of <0.05 were considered to indicate statistical significance.

### Ethics Approval

At the time of enrollment, written informed consent was obtained from all patients. The study protocol was approved by the institutional review board of Asan Medical Center (approval number 2019-0007). All the study data were deidentified and collected according to the approval of the institutional review board. There was no compensation for the patients’ participation. This study was registered on ClinicalTrials.gov (NCT03783481).

## Results

### Descriptive Statistics

Table 1 shows patients’ clinical characteristics. The 2 groups did not show significant differences in patient-level characteristics, except for the higher mean age at diagnosis in the intervention group than in the control group (46.8 vs 44.7 years; *P*=.02). We therefore controlled for age at diagnosis in multivariate analyses.

**Table 1:** Patients’ clinical and sociodemographic characteristics.

	Intervention (N=85)	Control (N=90)	*P* value
Age at diagnosis (years), mean (SD)	46.8 (6.2)	44.7 (5.7)	.02
Married, n (%)	78 (91.8)	73 (81.1)	.07
Education (bachelor’s degree or higher), n (%)	82 (96.5)	87 (96.7)	>.99
Employed, n (%)	43 (50.6)	33 (36.7)	.09
Comorbidity, n (%)	50 (58.8)	52 (57.8)	>.99
Episode of depression, n (%)	0 (0)	1 (1.1)	>.99
Previous chemotherapy, n (%)	24 (28.2)	25 (27.8)	>.99
Antihormonal therapy, n (%)	66 (77.6)	75 (83.3)	.45
Follow-up (weeks), median (SD)	14 (7.5)	16 (7.6)	.65

### Primary and Secondary Outcomes

Table 2 shows the results of our multivariate analyses, in which the intervention group had a significantly lower degree of moderate to severe distress during the 24-week period (B=–0.558; odds ratio [OR] e^(–0.558)=0.572; *P*<.001; number of collected data points 2016). The intervention group also had a significantly higher number of total weekly step counts as well (B=0.125; rate ratio 1.132; *P*<.001; number of collected data points 121).

**Table 2:** Multivariate analysis of moderate to severe distress (164 patients; 2016 data points; AIC^a^=2382.40) and weekly step counts (51 patients; 121 data points; AIC=147.33) for the entire 24-week study period.

	Moderate to severe distress			Weekly step counts	
	B (SE)	*P* value	B (SE)		*P* value
Intervention group (vs control group)	–0.558 (0.102)	<.001	0.125 (0.031)		<.001
Age at diagnosis	0.010 (0.010)	>.99	0.007 (0.003)		.01
Married	0.437 (0.148)	.003	–0.100 (0.049)		.04
Education (bachelor’s degree or higher)	1.452 (0.269)	<.001	—^b^		—
Employed	0.214 (0.112)	.06	–0.101 (0.032)		.002
Comorbidity	–0.050(0.108)	.64	–0.077 (0.032)		.02
Episode of depression	12.53 (312.10)	.97	—		—
Previous chemotherapy	0.033 (0.119)	.78	–0.057 (0.035)		.11
Antihormonal therapy	–0.829 (0.159)	<.001	–0.009 (0.041)		.83

^a^Akaike information criterion.

^b^Not available.

### Sensitivity Analyses

Table 3 shows the results of sensitivity analyses of the past 4 weeks, in which the intervention group had a lower degree of moderate to severe distress (B=–0.823; OR 0.439; *P*=.08; number of collected data points 121) and significantly higher number of weekly step counts (B=0.196; rate ratio 1.217; *P*=.02; number of collected data points 264).

In terms of mild to severe distress (Table 4), the intervention group exhibited significantly lower levels during the total study period (B=–0.731; OR 0.483; *P*<.001) and a trend toward lower levels in the past 4 weeks (B=–0.881; OR 0.414; *P*=.08). No harmful effect of the intervention was reported.

**Table 3:** Multivariate analysis of moderate to severe distress (51 patients; 121 data points; AIC^a^=147.33) and weekly step counts (70 patients; 264 data points; AIC=0.06) during the final 4 weeks.

	Moderate to severe distress			Weekly step counts	
	B (SE)	*P* value	B (SE)		*P* value
Intervention group (vs control group)	–0.823 (0.469)	.08	0.196 (0.086)		.02
Age at diagnosis	0.061 (0.046)	.19	0.001 (0.008)		.9
Married	1.267 (0.716)	.08	–0.093 (0.144)		.52
Education (bachelor’s degree or higher)	1.405 (1.587)	.38	—^b^		—
Employed	0.237 (0.491)	.63	–0.280 (0.093)		.003
Comorbidity	–0.666 (0.490)	.17	0.019 (0.095)		.84
Episode of depression	—	—	—		—
Previous chemotherapy	–0.374 (0.497)	.45	–0.037 (0.097)		.71
Antihormonal therapy	–2.289 (1.111)	.04	–0.141 (0.116)		.23

^a^Akaike information criterion.

^b^Not available.

**Table 4:** Multivariate analysis of mild to severe distress (Distress Thermometer score ≥3) for the total study period (164 patients; 2016 data points; AIC^a^=2047.8) and the final 4 weeks (51 patients; 121 data points; AIC=137.7).

	Total study period (weeks 1 to 24)			Past 4 weeks (weeks 21 to 24)	
	B (SE)	*P* value	B (SE)		*P* value
Intervention group (vs control group)	–0.731 (0.114)	<.001	–0.881 (0.496)		.08
Age at diagnosis	0.010 (0.011)	.35	0.042 (0.047)		.38
Married	0.477 (0.160)	.003	0.871 (0.675)		.2
Education (bachelor’s degree or higher)	0.718 (0.303)	.02	–14.86 (1520.29)		.99
Employed	0.126 (0.125)	.32	–0.020 (0.519)		.97
Comorbidity	–0.146 (0.120)	.22	–0.893 (0.513)		.08
Episode of depression	12.03 (312.10)	.97	—^b^		—
Previous chemotherapy	–0.024 (0.131)	.85	–0.659 (0.516)		.2
Antihormonal therapy	–0.848 (0.183)	<.001	–2.10 (1.11)		.06

^a^Akaike Information Criterion.

^b^Not available.

## Discussion

### Principal Results

This trial assessed the effect of app-based mobile community engagement on the mental health and physical activity of breast cancer survivors. Mobile community engagement led to a significantly lower level of moderate to severe mental distress and a higher level of physical activity, as evidenced by weekly step counts, for the 24-week study period. In the final 4 weeks, these effects remained significant in terms of increased physical activity and exhibited a trend toward lower levels of distress.

To the best of our knowledge, this study represents the first randomized, controlled investigation into the impact of participating in a peer community through mobile devices on the behaviors and distress levels of cancer survivors. Extensive research has been conducted to explore approaches for alleviating distress in this population, given their heightened susceptibility to psychological challenges. A meta-review of randomized controlled trials focusing on health interventions revealed a notable reduction in distress levels [28]. This review encompassed 29 randomized controlled trials involving a total of 3274 participants. The focus was on mindfulness-based interventions, which typically consisted of an average of 16.6 contact hours. These sessions were conducted by mindfulness instructors with relevant professional backgrounds. Another systematic review of telephone interventions addressing psychosocial distress in cancer survivors suggested these methods may be effective in reducing distress [29]. However, practicality must be considered when using these methods. Conventional interventions typically require trained professionals, education sessions, and assessment of instructor competence, which can be a strain on resources in busy practices where the focus is mainly on cancer treatments. Therefore, it is noteworthy that we found that engaging cancer survivors in a mobile app–based community was a resource-saving and effective intervention for reducing distress. It is true that an offline peer support group may offer more support compared to a web-based group. The objective of this study was to demonstrate that web-based communities not only provide effectiveness but also offer convenience in delivering support, specifically in enhancing physical activities and reducing mental distress. It is crucial to recognize that in certain circumstances where offline communities may not be accessible, such as during the COVID-19 pandemic, mobile communities can serve as an effective alternative for providing support to cancer survivors.

Several studies have investigated the effect of electronic health technology on managing distress in cancer survivors, most of which involved web-based interventions. One randomized controlled study that included 129 cancer survivors reported that a web-based stress management program was effective in improving their quality of life [30]. Other internet-based studies also showed the feasibility of those interventions in cancer survivors [31,32], which typically provide modules for education, strategies, and exercise. However, these web-based programs require 15 to 90 minutes per session to complete. While web-based programs have demonstrated effectiveness, they may pose practical challenges and have limited feasibility in real-world settings. In this study, participants in the intervention group were only required to access the mobile app once per week. Apart from that, they had the freedom to monitor their daily step counts and compare their rankings with other members of the community.

A systematic review has indicated that mobile app–based health interventions have the potential to encourage positive behaviors, such as physical activity and weight management, among the general population [33]. Previous studies used mobile app–based interventions as tools for providing information, self-monitoring, and feedback, which led to increased use of sunscreen [34], weight reduction [35,36], and changes in physical activity and sedentary behavior [12]. Although mobile phone–based interventions appear to improve healthy behaviors in the general population, studies on their use among cancer survivors are still limited. We previously conducted a prospective feasibility study, which showed the effect of an mHealth community on increasing physical activities and reducing mental distress in breast cancer survivors [24]. This study builds upon the previous endeavors by offering further evidence in support of using mobile technology to improve the quality of care for cancer survivors.

In this study, mobile community engagement led to significantly lower levels of distress during the total study period of 24 weeks. However, during the past 4 weeks, the reduction in moderate to severe distress through mobile community engagement fell short of having statistical significance (Table 3; *P*=0.08). This lack of statistical significance may be due to the relatively low number of data points (n=121) compared with the total period (n=2016), which was likely attributable to the decline in the reporting of the app-based questionnaire by the patients over time (Multimedia Appendix 2). In contrast, data on weekly step counts were automatically collected by the app, thereby demonstrating the feasibility of smartphone apps in collecting physical activity data of patients with cancer, as shown in our previous study [23].

The beneficial effect of mobile community engagement on mental distress and physical activity can be explained by several theories [37,38]. The theory of planned behavior posits that attitudes, norms, and behavioral control are key determinants that influence intentions and behaviors. Attitude reflects an individual’s personal viewpoint toward a specific behavior; subjective norms relate to their perception of others’ opinions about the behavior; and perceived behavioral control resembles self-efficacy and denotes an individual’s sense of control over their actions. In our study, patients in the intervention group exhibited favorable attitudes and subjective norms toward daily walking. Additionally, receiving feedback on their daily step counts and rankings may have enhanced their self-efficacy.

### Limitations

This study should be considered with the following limitations. First, this study is a single-centered study. Thus, the results of this trial may not be applicable in other settings. Second, we excluded patients undergoing chemotherapy and only included those who had completed preoperative chemotherapy. We applied these selection criteria considering that chemotherapy usually entails a range of adverse effects that could confound the assessment of the effect of mobile community engagement. Third, the data collection rate for distress decreased over time as it relied on self-reporting through the mobile app. Fourth, older patients were excluded in this study because it was not easy for them to perform the study protocols, such as checking app-based weekly distress thermometer questionnaires, based on which the primary outcome was. Although these patients were excluded, the results of our study can still be effective for older patients. Because the mobile community in this study does not require them to submit any reports. The app gathers participants’ daily activities automatically for these patients and gives feedback to enhance daily walking. Fifth, although walking is convenient compared to other forms of exercise and can be recommended to patients with breast cancer without significant burden, our study included walking as the only form of physical activity. Sixth, given the nature of step counts, which are reflective of individual behavioral patterns, it is deemed challenging for them to follow a normal distribution. Therefore, there is a limitation in our study regarding the potential for larger variance in the estimated coefficients. Last, the short follow-up period of the study does not provide evidence of long-lasting impact of peer community engagement on the mental distress and physical activity of breast cancer survivors.

### Generalizability

Cancer survivors are known to have mental distress and physical inactivity after cancer diagnosis [39,40]. For this reason, although this study was conducted on female breast cancer survivors, we believe that our results may be readily applicable to patients who have survived other types of cancer. In addition, smartphone penetration rates are high across all regions, and this study used a freely available activity tracker app. Therefore, our results may be recapitulated in studies using other types of mobile apps.

### Conclusions

Distress management and enhancing physical activity are critical issues for cancer survivors. Engagement in a mobile app–based peer community was a promising tool for reducing distress and encouraging walking activity in breast cancer survivors. Mobile technology can be effective in improving the level of care for cancer survivors.

## Appendix

Multimedia Appendix 1 Supplementary Figure 1. User interfaces of the WalkON app.

Multimedia Appendix 2 Supplementary Figure 2. Weekly number of patients who submitted the Distress Thermometer questionnaire on the mobile app.

Multimedia Appendix 3 CONSORT eHEALTH checklist (V 1.6.2).

## Abbreviations

DT: Distress Thermometer
mHealth: mobile health
OR: odds ratio
RR: rate ratio

## Data Availability

The anonymously processed data set used in this study can be shared upon reasonable request with the corresponding author. Any interested party should submit an appropriate proposal, analysis plan, and protocols for review and approval by the authors. Subsequently, the materials will be submitted for approval to the institutional review board of Asan Medical Center (Seoul, Republic of Korea). Once approved, the data will be made available. This study was presented at the San Antonio Breast Cancer Symposium on December 9, 2022.
