# Supplementary figures and images for: The Impact of a Mobile Support Group on Distress and Physical Activity in Breast Cancer Survivors: Randomized, Parallel-Group, Open-Label, Controlled Trial
Source: J Med Internet Res. 2023 Aug 7;25:e47158. doi: 10.2196/47158 (PMC10442738; doi:10.2196/47158)

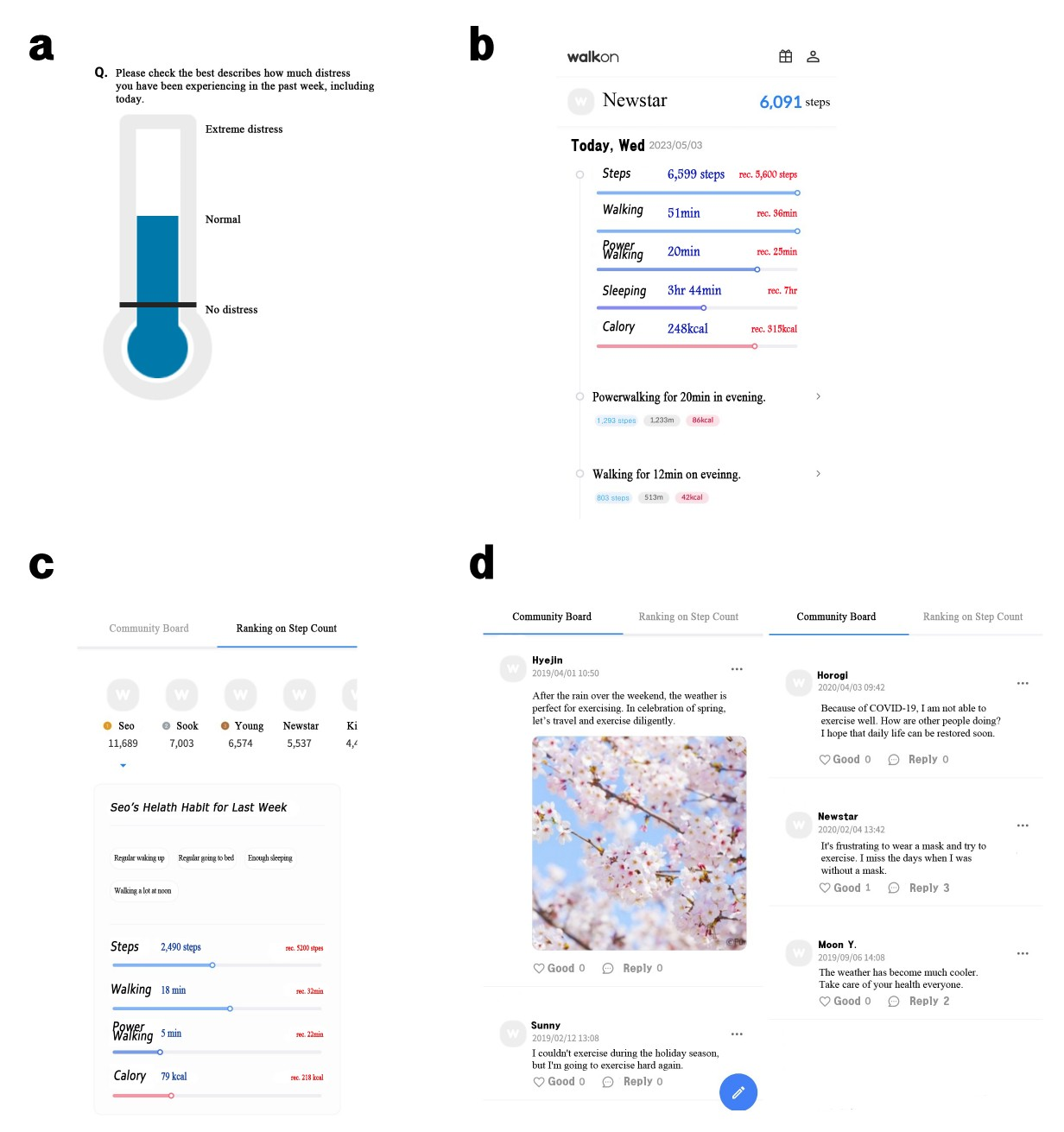

Supplement: Multimedia Appendix 1 [file jmir_v25i1e47158_app1.png]

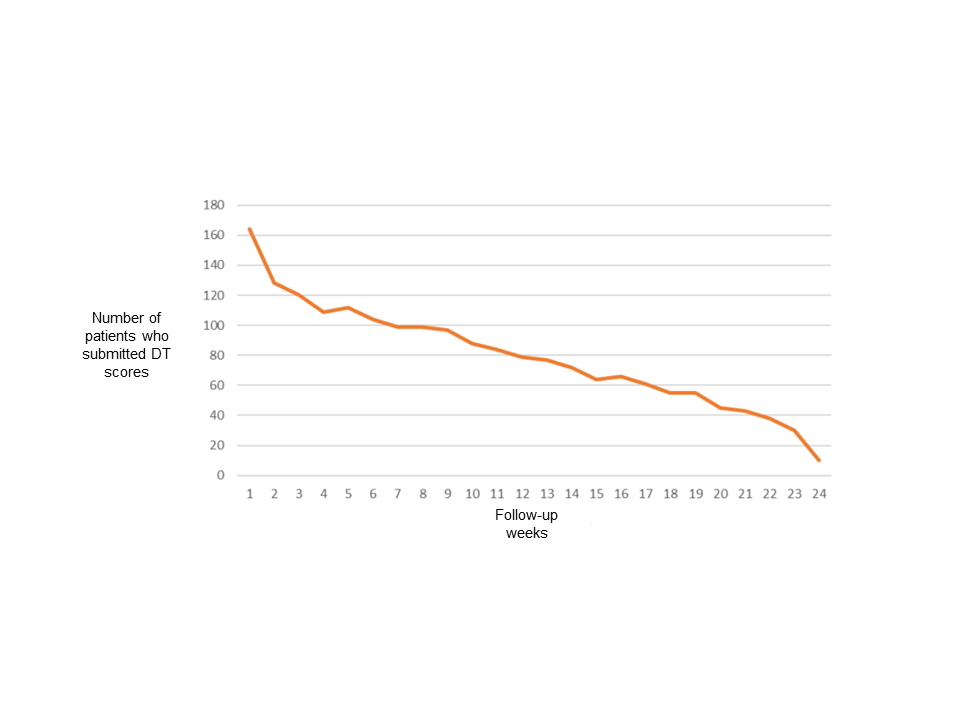

Supplement: Multimedia Appendix 2 [file jmir_v25i1e47158_app2.png]
